# Gastronet survey on the use of one- or two-person technique for colonoscopy insertion

**DOI:** 10.1186/1471-230X-11-73

**Published:** 2011-06-14

**Authors:** Geir Hoff, Moritz Volker, Michael Bretthauer, Lars Aabakken, Ole Høie, Thomas deLange, Ingrid Berset, Øystein Kjellevold, Tom Glomsaker, Gert Huppertz-Hauss, Ove Lange, Per Sandvei

**Affiliations:** 1 Dept of Medicine, Telemark Hospital, 3710 Skien, Norway; 2 Cancer Registry of Norway, Oslo University Hospital, Montebello, 0304 Oslo, Norway; 3 Oslo University Hospital Rikshospitalet, Dept of Medicine, 0027 Oslo, Norway; 4 Dept of Medicine, Sørlandet Hospital Arendal, 4809 Arendal, Norway; 5 Dept of Medicine, Bærum Hospital Vestre Viken HF, 1309 Rud, Norway; 6 Dept of Medicine, Helse Sunnmøre HF, Ålesund Hospital, 6026 Ålesund, Norway; 7 Telemark Hospital Kragerø, 3770 Kragerø, Norway; 8 Dept of Surgery, Stavanger University Hospital, 4068 Stavanger, Norway; 9 Dept of Medicine, Telemark Hospital, 3710 Skien, Norway; 10 Dept of Medicine, Molde Hospital, Dept of Medicine, 6407 Molde, Norway; 11 Dept of Medicine, Østfold Hospital Trust, 1603 Fredrikstad, Norway

## Abstract

**Background:**

Usually, colonoscopy insertion is performed by the colonoscopist (one-person technique). Quite common in the early days of endoscopy, the assisting nurse is now only rarely doing the insertion (two-person technique). Using the Norwegian national endoscopy quality assurance (QA) programme, Gastronet, we wanted to explore the extent of two-person technique practice and look into possible differences in performance and QA output measures.

**Methods:**

100 colonoscopists in 18 colonoscopy centres having reported their colonoscopies to Gastronet between January and December 2009 were asked if they practiced one- or two-person technique during insertion of the colonoscope. They were categorized accordingly for comparative analyses of QA indicators.

**Results:**

75 endoscopists responded to the survey (representing 9368 colonoscopies) - 62 of them (83%) applied one-person technique and 13 (17%) two-person technique. Patients age and sex distributions and indications for colonoscopy were also similar in the two groups. Caecal intubation was 96% in the two-person group compared to 92% in the one-person group (p < 0.001). Pain reports were similar in the groups, but time to the caecum was shorter and the use of sedation less in the two-person group.

**Conclusion:**

Two-person technique for colonoscope insertion was practiced by a considerable minority of endoscopists (17%). QA indicators were either similar to or better than one-person technique. This suggests that there may be some beneficial elements to this technique worth exploring and try to import into the much preferred one-person insertion technique.

## Background

Painless, complete colonoscopy is a service in increasing demand as colorectal cancer screening is expanding in many countries. Sedation may partly solve the problem at an additional expense on resources [1]. A range of technological novelties may also contribute to ease insertion and reduce pain and discomfort [2,3]. Still, the skills of the endoscopist remain the crucial element to provide your patients with a painless, complete inspection of the colorectal mucosa [4]. In the early days of colonoscopy with long, stiff and cumbersome colonoscopes it was quite usual for the endoscopist to have a nurse to do the physical insertion of the endoscope directed by the endoscopist while inspecting the lumen. This is still practiced in some countries although the rationale for doing this in the 21^st ^century seems unclear. The present study is based on data from a national programme for quality assurance of colonoscopy and a survey to explore the extent of two-person colonoscopy insertion technique and evaluate the performance of one-person compared to two-person technique.

## Methods

**Figure 1:**
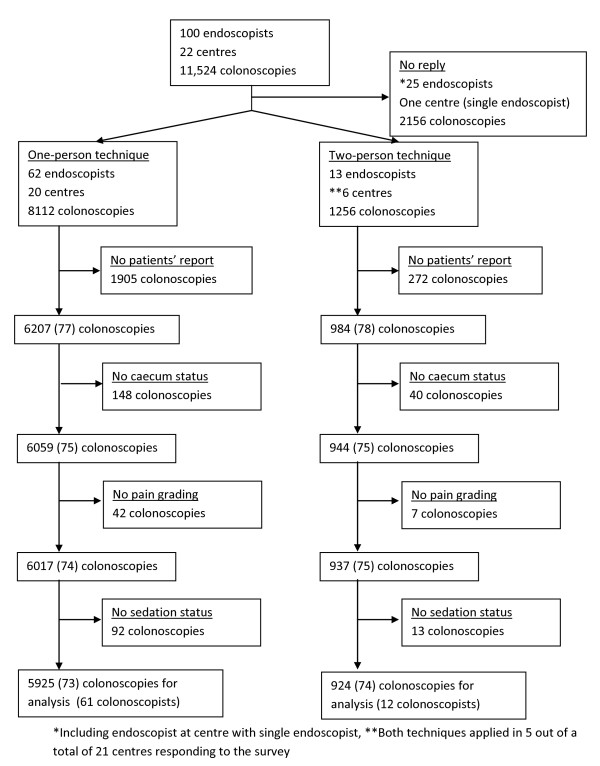
**Flow chart displaying endoscopists, centres and colonoscopies in the survey (percent)**.

Gastronet is a quality assurance programme for gastrointestinal endoscopy based in Norway by permission from the Data Inspectorate of Norway [5]. In February 2010, 100 colonoscopists from 18 hospitals on the Gastronet mailing-list and having performed colonoscopy in 2009 were asked by e-mail whether they applied one- or two-person technique for colonoscopy insertion, i.e. inserted the endoscope themselves or by nurse assistance. Colonoscopists were categorized into "one-person technique" or "two-person technique". One-person technique was defined by only the endoscopist personally advancing the endoscope during insertion. Two-person technique was defined as colonoscopy performed with a nurse or assistant actively advancing the colonoscope at any stage of insertion. Colonoscopy performance indicators (including caecum intubation rate and time, withdrawal time and the rate of detection of polyps ≥ 5 mm and patient-reported pain) were registered from January to December 2009 for both categories of colonoscopists. Only colonoscopies with returned patients' questionnaires were included in the analysis. Further exclusions from the analyses were colonoscopies not reporting caecal intubation status, whether sedation was used or not and patient's pain score (Figure 1). Patients' questionnaires were filled in at home on the day after the examination and returned directly to the coordinating Gastronet secretariat (not to the responsible endoscopist or hospital). Patients' replies included categorization of pain experienced as "no pain", "slight pain", "moderate pain" or "severe pain".

Withdrawal time has been used as a surrogate endpoint for thoroughness of inspection. Since polyp detection triggers time-consuming tissue sampling procedures, we included withdrawal times for purely visual diagnostic colonoscopies in a logistic regression analysis to identify a possible independent role of endoscopist category in polyp detection.

Personal information on patients in the database was restricted to gender and 5-year age categories (not exact age in years). Age of endoscopist was used as surrogate for experience.

### Statistics

We presented the results as univariate analyses according to application of one- or two-person intubation method with Chi square statistical analysis for categorical data and Student's T-test for continuous variables (age of endoscopists). A backward logistic regression analysis was performed to explore independent roles of withdrawal time and endoscopist technique category with regard to polyp detection. Statistical significance was defined as p < 0.05 using two-sided tests and the statistical package SPSS 15.0 was used (SPSS Inc., Illinois, USA).

### Ethical considerations

In line with Norwegian law, the Regional Ethics Committees waived the needs for approval of the study because it was performed within the Gastronet quality assurance program, which has been approved by the Norwegian Data Inspectorate.

## Results

75 of 100 colonoscopists responded to the survey - 62 of them (83%) applying one-person technique and 13 (17%) two-person technique (Figure 1). 1256 (13%) out of 9368 colonoscopies were reported as two-person colonoscopies.

The median patient 5-year age category was 61-65 years. The age and sex distributions and the indications for colonoscopy were comparable in the two groups (table 1).

**Table 1:** Demographics of patients and indications for colonoscopy (percent)

	One-person technique (n = 5925)	Two-person technique (n = 924)	p-value
*Gender			

Male	2670 (45)	441 (48)	0.15
	
Female	3228 (55)	481 (52)	

*Age category			

< 61 years	2622 (44)	410 (45)	
	
61-65 years	911 (15)	154 (17)	0.55
	
> 65 years	2367 (40)	357 (39)	

Indications			

Symptoms	3728 (63)	594 (64)	
	
Controls (polyps, CRC, IBD)	1315 (22)	227 (25)	0.02
	
Screening (including family predisposition)	480 (8.1)	54 (5.8)	
	
Other, unspecified	402 (6.8)	49 (5.3)	

29 colonoscopies not reported with gender of patient and 28 without age category

CRC = colorectal cancer

IBD = chronic inflammatory bowel disease

After exclusions (Figure 1), the 61 remaining endoscopists in the one-person group reported on average 26% more colonoscopies per endoscopist than the 12 endoscopists in the two-persons group - 97 and 77 colonoscopies per endoscopist, respectively. The mean age of endoscopists was 51 years (95% confidence interval 48-54 years) in the one-person group and 54 years (95% confidence interval 48-61 years) in the two-persons group (p = 0.34).

For colonoscopies performed with one-person technique, the caecal intubation rate was 92% compared to 96% for two-person technique (p < 0.001), but one-person technique appeared to be associated with better polyp detection rates than two-person technique in univariate analysis (table 2). A logistic regression analysis showed, however, that this was independently associated with longer mean withdrawal time in the one-person group (7.70 minutes (95% CI 7.54-7.85)) compared to the two-person group (5.39 minutes (95% CI 5.14-5.65)) and not associated with endoscopist insertion technique (table 3). Some endoscopists in the two-person group converted occasionally to one-person technique and vice versa during phases of insertion problems.

**Table 2:** Patients' and endoscopists' report on colonoscopies performed with one- or two-person insertion technique (percent)

	One-person technique (n = 5925)	Two-person technique (n = 924)	p-value
Intubation rate			

Caecum reached	5460 (92)	889 (96)	
	
Caecum not reached	370 (6.2)	30 (3.2)	
	
Caecal intubation not intended	95 (1.6)	5 (0.5)	< 0.001

Detection of polyps ≥ 5 mm	1336 (23)	170 (18)	0.005

Intubation time (minutes, mean, 95%CI)	13.6 (13.4-13.9)	9.5 (9.0-10.0)	< 0.001

Withdrawal time (mean, 95%CI)	7.70 (7.54-7.85)	5.39 (5.14-5.65)	< 0.001

On-demand sedation/analgesics			

No sedation/analgesia	4250 (72)	771 (83)	< 0.001
	
Sedation/analgesia given	1675 (28)	153 (17)	

Pain during colonoscopy			

No pain	1659 (28)	265 (29)	
	
Slight pain	2407 (41)	394 (43)	0.32
	
Moderate pain	1119 (19)	167 (18)	
	
Severe pain	740 (13)	98 (11)	

**Table 3:** Logistic regression analysis showing that the increased odds ratio (OR) for detecting polyps 5 mm or larger with the one-person technique is due to longer withdrawal time.

	Unadjusted OR (95% confidence interval)	p-value	Adjusted OR (95% confidence interval)	p-value
Male sex	1.00 (reference)		1.00 (reference)	

Female sex	0.73 (0.66-0.81)	< 0.001	0.85 (0.75-0.96)	0.008

Age (5-year categories)	1.15 (1.13-1.17)	< 0.001	1.15 (1.12-1.18)	< 0.001

One- person technique	1.00 (reference)		1.00 (reference)	

Two-person technique	0.77 (0.65-0.93)	0.005	1.18 (0.97-1.43)	0.095

Withdrawal time	1.14 (1.13-1.15)	< 0.001	1.15 (1.14-1.16)	< 0.001

Intubation time was shorter and the use of on-demand sedation/analgesia was less for two-person compared to one-person technique - both comparisons highly significant (p < 0.001) (table 2). Pain reports were very similar for patients examined with one- and two-person techniques (table 2).

In 6849 colonoscopies performed by endoscopists responding to the survey, caecum was reached in 6349 (93%) compared to 1391 (94%) out of 1476 colonoscopies by non-respondents (p = 0.03). Examinations performed by non-respondents were more often with sedation (601 (41%)) than examinations by respondents (1828 (27%)) (p < 0.001). Similarly, patients of non-respondents reported more often moderate or severe pain (522 (35%)) than patients of respondents (2125 (31%)) (p = 0.001).

## Discussion

This survey showed that two-person technique for colonoscope insertion is practiced by a considerable minority of endoscopists in Norway (17%) covering 13% of colonoscopies. Endoscopists using one-person technique performed approximately 25% more colonoscopies per year than those using two-person technique. Reasons for this may be many - one being limited access to nurse assistance. Endoscopist age has been used by others as surrogate indicator for experience, showing a correlation with caecal intubation rate [6]. Others also reporting from routine clinical practice have not found a correlation between endoscopist experience and intubation rates [7]. Unexpectedly, the age distributions for endoscopists in our two study groups were very similar, suggesting that the practice of two-person technique is not restricted to endoscopists facing imminent retirement, but is also adopted by new generations in centres used to this method. There are variations within the present definition of two-person technique as there are for one-person technique. In the present study two-person technique was practiced with a nurse assistant advancing the endoscope during the whole or part of the insertion phase of the examination. All endoscopists practiced one-person withdrawal technique. The risk of extra use of nurse resources with two-persons technique may be partly compensated by a shorter intubation time (9.5 minutes compared to 13.6 using one-person technique) and less frequent use of sedation (17% compared to 28%) with similar frequencies of pain categories reported in both groups. It was surprising to find that even caecal intubation rates were better in the two-person group - 96% compared to 92% in the one-person group. The only apparent advantage of one-person technique was a slightly higher detection rate for polyps measuring 5 mm or more (23% compared to 18%), but logistic regression analysis disclosed that this could be explained by longer withdrawal time in the one-person group. With similar sex and age distribution and comparable indications for the colonoscopy there was no indication of patient selection between the groups. Some 80% of polyps > 5 mm are adenomas [8,9] and it has been shown that an adenoma detection rate of less than 20% at age 40-66 years substantially increases the risk of colorectal cancer within a 3-10 year perspective [10]. A 5% difference in the present polyp detection rates around 20% may therefore not be negligible, but it was reassuring to find that this was related to differences in withdrawal times between the groups and not to the present technique categories of colonoscopists.

As far as we know, performance comparisons of one- and two-person insertion techniques have not been reported before. The reasons for largely better performance in the two-person group are obscure and presently speculative. Shorter intubation time may be a team-work effect securing focus on a common goal and avoid the time spent as a time for "peace and rest" from on-call demands for the endoscopist. A stronger involvement of a nurse may also have a favourable influence on patients' pain perception and comfort.

A strength of the present study was the collection of performance data as part of a national quality assurance programme (Gastronet) irrespective and independent of centre or endoscopist compliance to the survey. This allowed access to quality indicator results from non-respondents showing that the present survey may not be representative for the Norwegian situation expressed by data in the quality assurance programme. Non-respondents performed colonoscopies with more pain and more frequent use of on-demand sedation while intubation rates showed only a one percent difference. This demonstrates that national quality assurance programmes like Gastronet are well suited for this type of survey to uncover potential problems of selection bias. There is no indication that one- or two-person techniques should be over- or under-represented among respondents, but this possibility cannot be excluded. Ideally, this study should have been a trial randomising endoscopist trainees to training with one- or two-person insertion technique and subsequent prospective registration of post-training colonoscopies, but this was considered unfeasible due to economic and staffing restraints. Also, we were not able to determine which of the two techniques was consistently used for each individual examination, but applied a categorical technique characteristic of the colonoscopist performing each examination.

Colonoscopy courses generally do not consider training in two-person techniques, but underline the importance of the endoscopist having full control and continuous feel of resistance to optimize combined rotation, angulation, straightening and advancement of the endoscope. There is no reason to change this strategy. Still, it is intriguing to find that a method considered by many as archaic represents colonoscopy performance characteristics that are increasingly sought - higher intubation rates, less sedation without compromising patients' report on pain and less time spent on intubation. The reasons for this are unclear. Further studies are needed to confirm our findings and to explore possible reasons for the differences observed. This should include qualitative studies to explore explanatory hypotheses. Possible advantageous elements of two-person technique should be sought and considered adopted in the prevailing training of one-person technique. In the meantime, there is no reason to look down on endoscopists practicing two-person technique, but rather try to extract some hidden secrets of their trade to be adopted into the much preferred one-person technique.

## Conclusion

The present study showed that the two-person colonoscope insertion technique may represent unrevealed qualities worth looking into to improve colonoscopy services.

## Competing interests

The authors declare that they have no competing interests.

## Authors' contributions

GH initiated the study, was responsible for the statistics and drafted the manuscript. VM, MB, LA, OH, TdL, IB, ØK, TG, GHH, OL and PS contributed with acquisition of data, interpretation of results, critical revision of the manuscript and approval of submission for publication. All authors read and approved the final manuscript.

## Pre-publication history

The pre-publication history for this paper can be accessed here:

http://www.biomedcentral.com/1471-230X/11/73/prepub
